# Supplementary material for: Functional safeguards for conservation: Identifying climate change refugia for frugivorous and nectarivorous birds in a degraded area of Colombia
Source: PLoS One. 2025 May 27;20(5):e0321817. doi: 10.1371/journal.pone.0321817 (PMC12111607; doi:10.1371/journal.pone.0321817)
Supplement: S1 Figure — (PDF) [file pone.0321817.s001.pdf]

Functional safeguards for conservation: identifying climate change refugia for frugivorous and nectarivorous birds in a degraded area of Colombia

Fausto Sáenz-Jiménez, María Alejandra Parrado-Vargas, José F. González-Maya & Juan Emiro Carvajal-Cogollo

SUPPORTING INFORMATION
S1 FIGURE

Supporting Information S1 Fig. Location of the study area (Magdalena province) in Colombia and South America

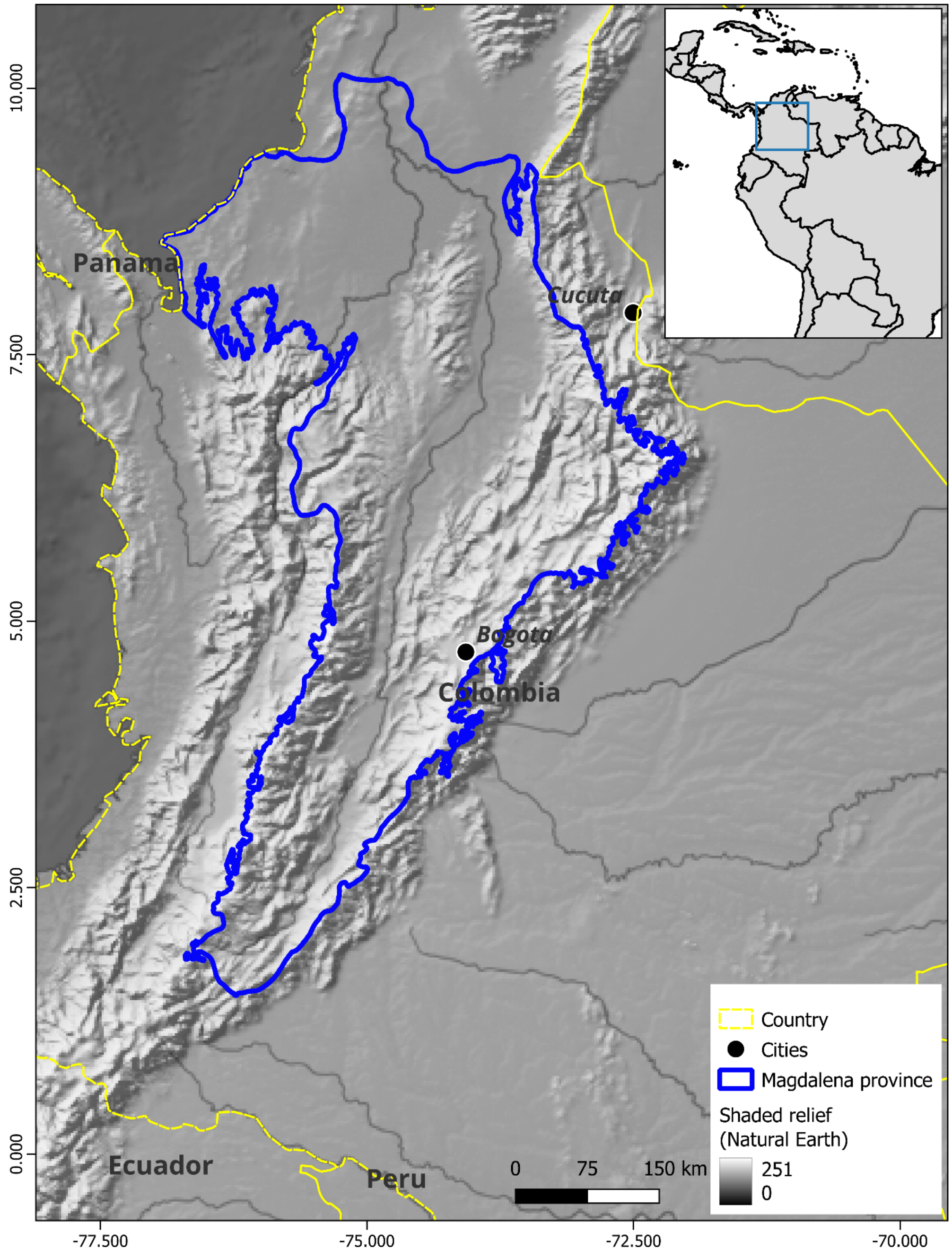

Functional safeguards for conservation: identifying climate change refugia for frugivorous and nectarivorous birds in a degraded area of Colombia

Fausto Sáenz-Jiménez, María Alejandra Parrado-Vargas, José F. González-Maya & Juan Emiro Carvajal-Cogollo

SUPPORTING INFORMATION
S2 FIGURE

Supporting Information S2 Fig. Correlation matrix plots for bird functional groups in the Colombian Magdalena biogeographic Province.

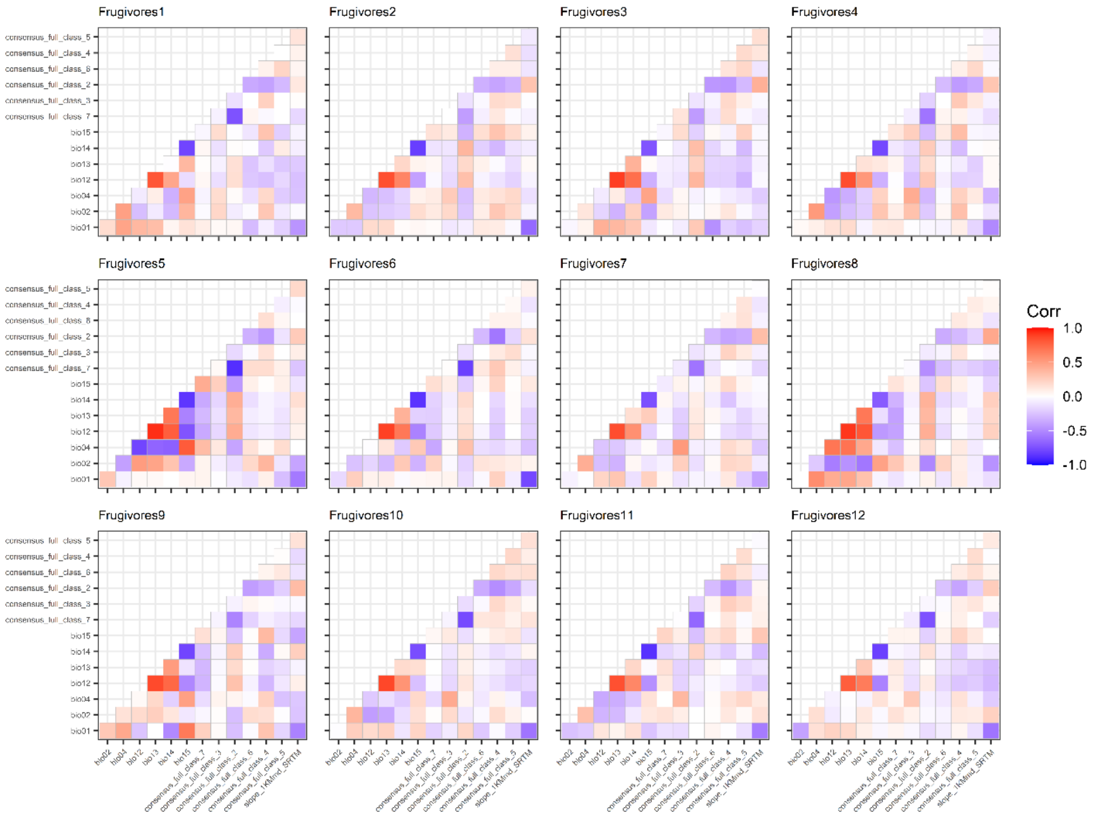

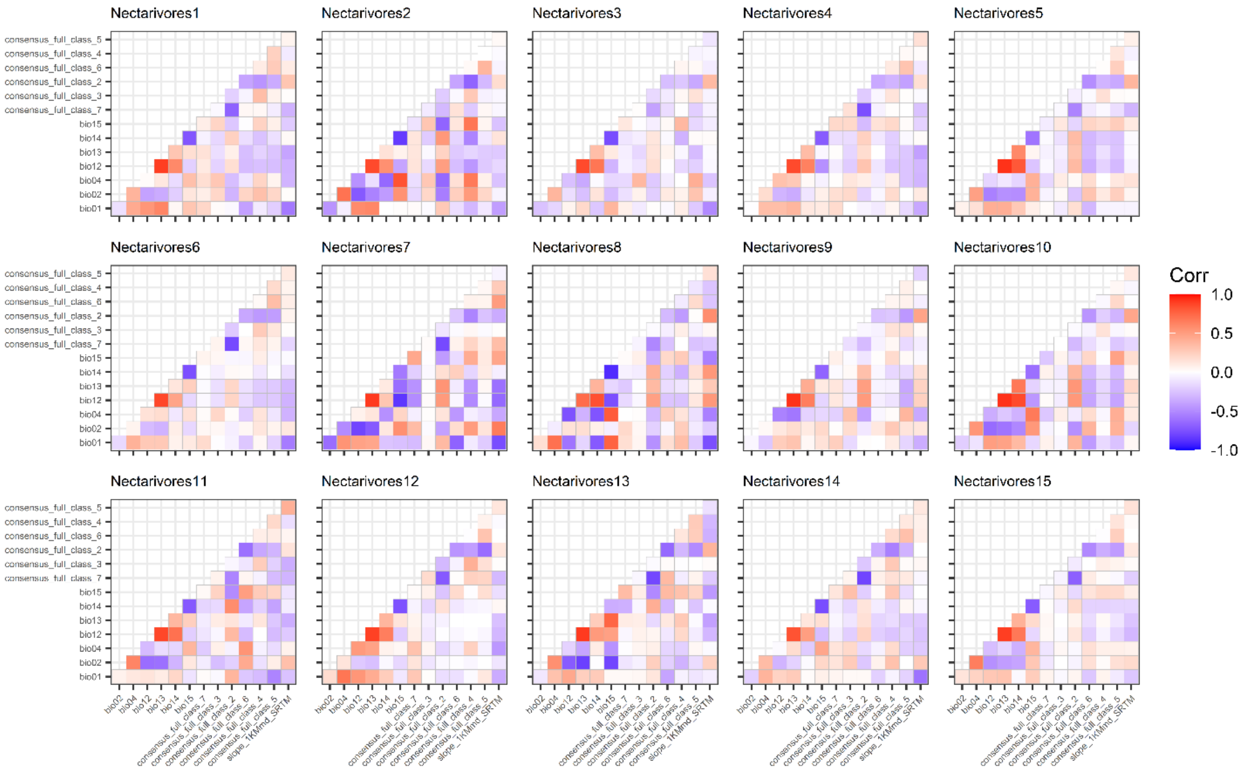
Nectarivores1
Nectarivores2
Nectarivores3
Nectarivores4
Nectarivores5
Nectarivores6
Nectarivores7
Nectarivores8
Nectarivores9
Nectarivores10
Nectarivores11
Nectarivores12
Nectarivores13
Nectarivores14
Nectarivores15
Corr
1.0
0.5
0.0
-0.5
-1.0
consensus_full_class_5
consensus_full_class_4
consensus_full_class_6
consensus_full_class_2
consensus_full_class_3
consensus_full_class_7
bio15
bio14
bio13
bio12
bio04
bio02
bio01
slope_1KMmd_SRTM

Functional safeguards for conservation: identifying climate change refugia for frugivorous and nectarivorous birds in a degraded area of Colombia

Fausto Sáenz-Jiménez, María Alejandra Parrado-Vargas, José F. González-Maya & Juan Emiro Carvajal-Cogollo

SUPPORTING INFORMATION
S1 FIGURE

Supporting Information 2 (S1 Figure). Exploration of traits for the frugivorous functional groups.

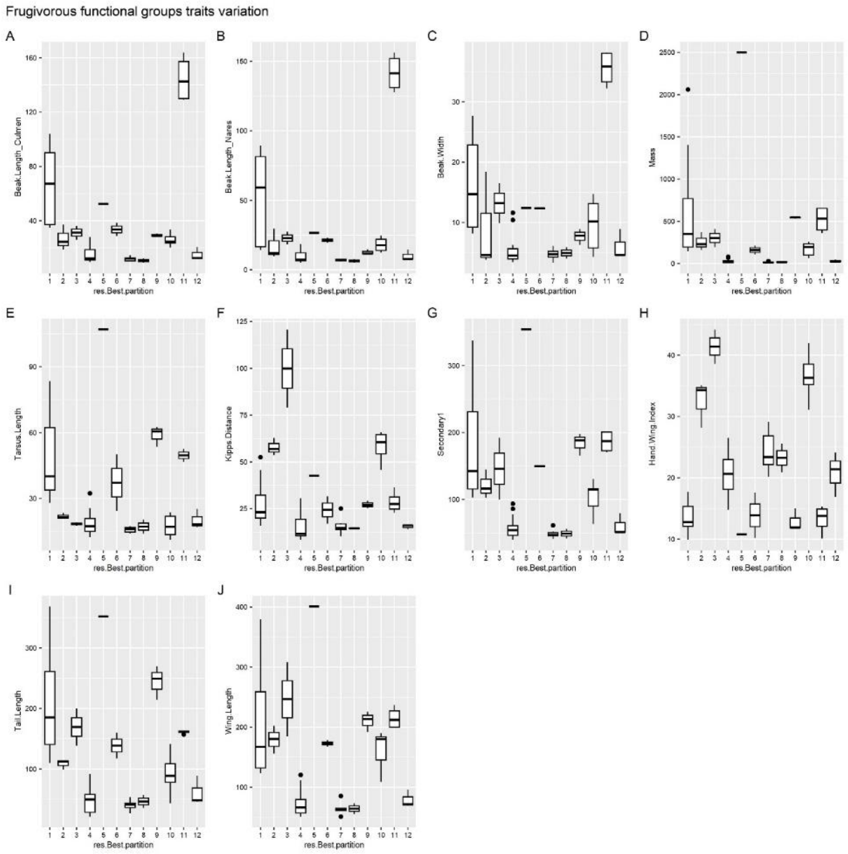

Boxplot of trait variability for functional groups of frugivorous birds.

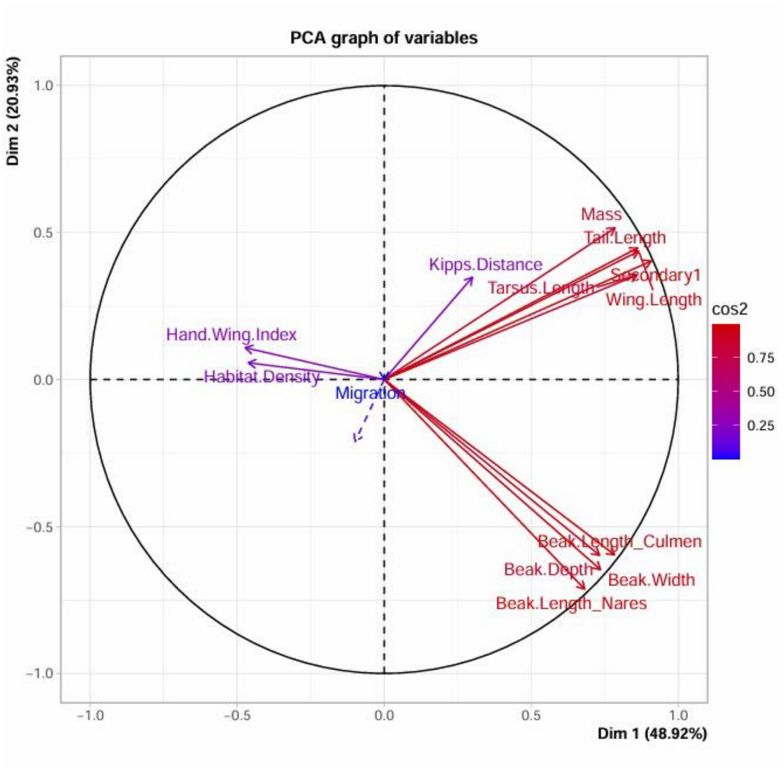

PCA ordination of trait variability for functional groups of frugivorous birds.

Functional safeguards for conservation: identifying climate change refugia for frugivorous and nectarivorous birds in a degraded area of Colombia

Fausto Sáenz-Jiménez, María Alejandra Parrado-Vargas, José F. González-Maya & Juan Emiro Carvajal-Cogollo

SUPPORTING INFORMATION
S2 FIGURE

Supporting Information 3 (S2 Figure). Exploration of traits for the nectarivorous functional groups.

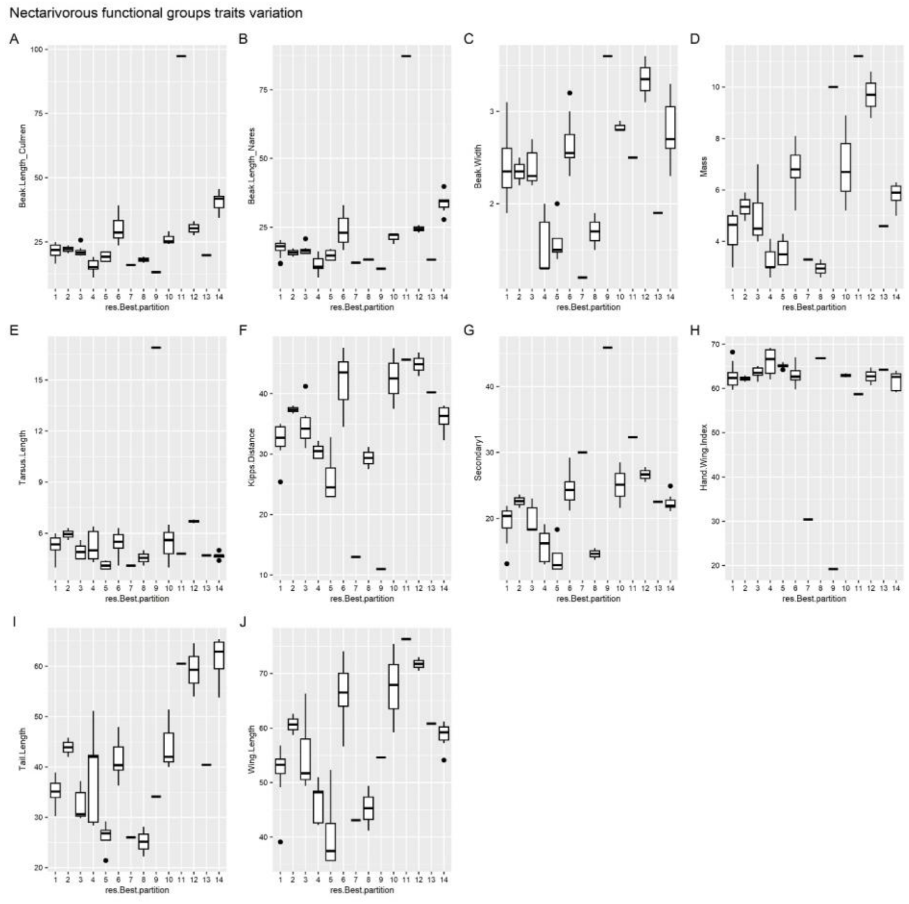

Boxplot of trait variability for functional groups of nectarivorous birds.

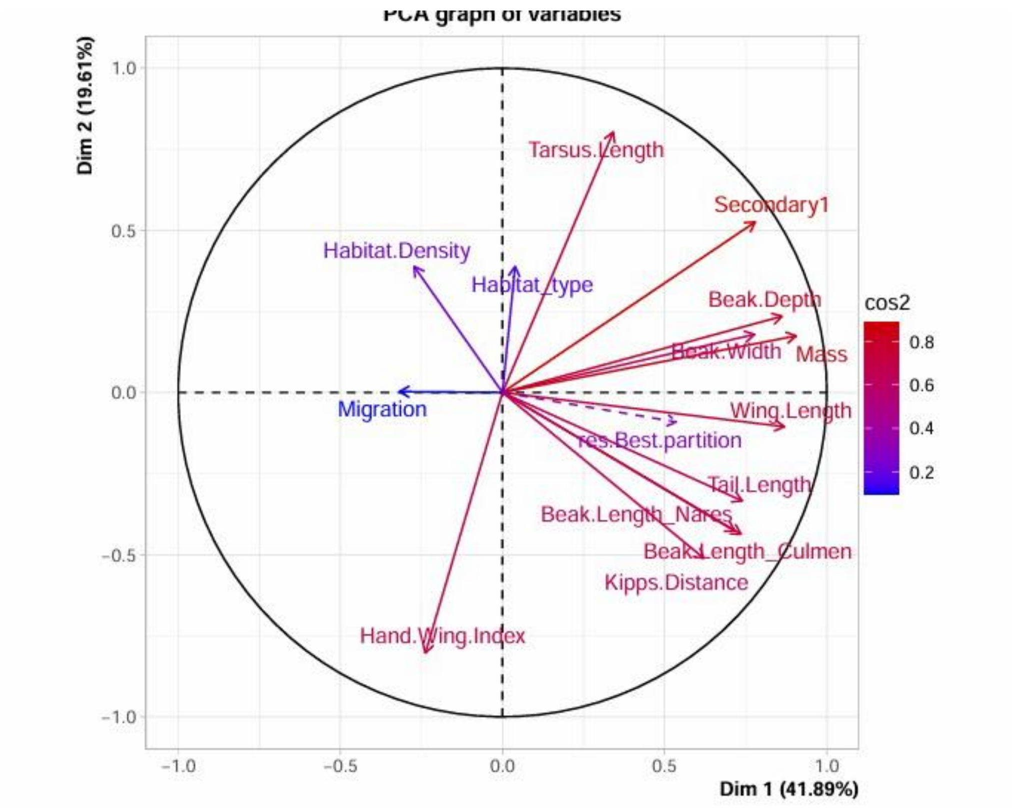

PCA ordination of trait variability for functional groups of nectarivorous birds.

Functional safeguards for conservation: identifying climate change refugia for frugivorous and nectarivorous birds in a degraded area of Colombia

Fausto Sáenz-Jiménez, María Alejandra Parrado-Vargas, José F. González-Maya & Juan Emiro Carvajal-Cogollo

SUPPORTING INFORMATION
S1 TABLE

Supporting Information 1 (S1 Table). Description of frugivorous and nectarivorous functional groups composition and description of traits.

Frugivorous functional group	Species	Traits description
Group 1	*Aburria aburri*<br>*Andigena nigrirostris*<br>*Aulacorhynchus albivitta*<br>*Aulacorhynchus haematopygus*<br>*Aulacorhynchus prasinus*<br>*Chamaepetes goudotii*<br>*Penelope argyrotis*<br>*Penelope montagnii*<br>*Penelope purpurascens*<br>*Pteroglossus azara*<br>*Pteroglossus torquatus*	Lifestyle: Insessorial (species spends much of the time perching above the ground, in branches of trees or on other raised substrates).<br>Habitat type: Dense Forest<br>Mass: 144-2060 gr.<br>Beak length (Culmen): 34-104 mm<br>Beak width: 8.2-27.7 mm.<br>Wing lenght: 123.5-379.7 mm.<br>Secondary feather length: 102.9-337.6 mm
Group 2	*Amazona amazonica*<br>*Patagioenas cayennensis*<br>*Patagioenas subvinacea*	Lifestyle: Insessorial (species spends much of the time perching above the ground, in branches of trees, or on other raised substrates).<br>Habitat type: Semi-open and open habitats<br>Mass: 99.2-114.4 gr.<br>Beak length (Culmen): 18.8-37.1 mm<br>Beak width: 3.8-18.4 mm.<br>Wing length: 156.3-202.2 mm.<br>Secondary feather length: 102.7-144.8 mm
Group 3	*Psittacara wagleri*<br>*Steatornis caripensis*	Lifestyle: Insessorial (species spends much of the time perching above the ground, in branches of trees, or on other raised substrates).<br>Habitat type: Dense Forest<br>Mass: 194-408 gr.<br>Beak length (Culmen): 26.1-36.3 mm<br>Beak width: 9.9-16.5 mm.<br>Wing length: 184.8-308.3 mm.<br>Secondary feather length: 99.5-192.1 mm
Group 4	*Ceratopipra erythrocephala*<br>*Corapipo leucorrhoa*<br>*Eubucco bourcierii*<br>*Euphonia minuta*	Lifestyle: Insessorial (species spends much of the time perching above the ground, in branches of trees, or on other raised substrates).<br>Habitat type: Dense Forest

Frugivorous functional group	Species	Traits description
	Legatus leucophaius<br>*Lepidothrix velutina*<br>*Machaeropterus striolatus*<br>*Manacus manacus*<br>*Mionectes oleagineus*<br>*Tangara arthus*<br>*Tangara gyrola*<br>*Tityra inquisitor*<br>*Tityra semifasciata*<br>*Turdus leucomelas*	Mass: 194-408 gr.<br>Beak length (Culmen): 9.7-26.3 mm<br>Beak width: 3.5-11.6 mm.<br>Wing length: 50.8-120.5 mm.<br>Secondary feather length: 39.5-93.4 mm
Group 5	*Crax alberti*	Lifestyle: Terrestrial (species spends majority of its time on the ground).<br>Habitat type: Dense Forest<br>Mass: 2499 gr.<br>Beak length (Culmen): 52 mm<br>Beak width: 12 mm.<br>Wing length: 401 mm.<br>Secondary feather length: 354mm
Group 6	*Cyanocorax affinis*<br>*Querula purpurata*	Lifestyle: Insessorial (species spends much of the time perching above the ground, in branches of trees, or on other raised substrates).<br>Habitat type: Dense Forest<br>Mass: 107-212 gr.<br>Beak length (Culmen): 28.8-38.4 mm<br>Beak width: 12.2-12.5 mm.<br>Wing length: 167.8-178.2 mm.<br>Secondary feather length: 148.7-150.7 mm.
Group 7	*Dacnis egregia*<br>*Euphonia fulvicrissa*<br>*Euphonia laniirostris*<br>*Euphonia xanthogaster*<br>*Pseudodacnis hartlaubi*<br>*Stilpnia cyanicollis*<br>*Tersina viridis*	Lifestyle: Insessorial (species spends much of the time perching above the ground, in branches of trees, or on other raised substrates).<br>Habitat type: Semi-open habitats<br>Mass: 107-212 gr.<br>Beak length (Culmen): 10.1-14.5 mm<br>Beak width: 3.3-6.1 mm.<br>Wing length: 51.2-85.4 mm.<br>Secondary feather length: 40.5-61.1 mm.
Group 8	*Euphonia concinna*<br>*Stilpnia vitriolina*	Lifestyle: Insessorial (species spends much of the time perching above the ground, in branches of trees, or on other raised substrates).<br>Habitat type: Semi-open habitats (shrubland, woodland)<br>Mass: 10.3-23 gr.<br>Beak length (Culmen): 9-12.2 mm<br>Beak width: 4-5.9 mm.<br>Wing length: 55.7-73.1 mm.<br>Secondary feather length: 41.4-56.1 mm.
Group 9	*Ortalis columbiana*<br>*Ortalis garrula*<br>*Ortalis guttata*	Lifestyle: Insessorial (species spends much of the time perching above the ground, in branches of trees, or on other raised substrates).<br>Habitat type: Dense shrubland<br>Mass: 532-547.7 gr.<br>Beak length (Culmen): 28.2-30.7 mm<br>Beak width: 6.3-8.9 mm.<br>Wing length: 192.3-225.6 mm.<br>Secondary feather length: 165.6-197.9 mm.
Group 10	*Patagioenas plumbea*<br>*Patagioenas speciosa*<br>*Pionus chalcopterus*<br>*Pionus tumultuosus*<br>*Pyrrhura calliptera*	Lifestyle: Insessorial (species spends much of the time perching above the ground, in branches of trees, or on other raised substrates).<br>Habitat type: Dense Forest<br>Mass: 65-258.5 gr.

Frugivorous functional group	Species	Traits description
	Touit dilectissimus	Beak length (Culmen): 20.2-33.5 mm<br>Beak width: 4.3-14.7 mm.<br>Wing length: 109-189.8 mm.<br>Secondary feather length: 63.2-130.6 mm.
Group 11	*Ramphastos ambiguus*<br>*Ramphastos sulfuratus*<br>*Ramphastos tucanus*<br>*Ramphastos vitellinus*	Lifestyle: Insessorial (species spends much of the time perching above the ground, in branches of trees, or on other raised substrates).<br>Habitat type: Dense Forest<br>Mass: 409.7-659.6 gr.<br>Beak length (Culmen): 20.2-33.5 mm<br>Beak width: 4.3-14.7 mm.<br>Wing length: 199-237 mm.<br>Secondary feather length: 170.3-201 mm
Group 12	*Saltator maximus*<br>*Stilpnia larvata*<br>*Tangara inornata*	Lifestyle: Insessorial (species spends much of the time perching above the ground, in branches of trees, or on other raised substrates).<br>Habitat type: Semi-open habitats (forest)<br>Mass: 409.7-659.6 gr.<br>Beak length (Culmen): 11.8-20.7 mm<br>Beak width: 4.6-8.9 mm.<br>Wing length: 69.3-96.1 mm.<br>Secondary feather length: 51.2-79.2 mm.

Nectarivorous functional group	Species	Traits description
Group 1	*Adelomyia melanogenys*<br>*Amazilia tzacatl*<br>*Haplophaedia aureliae*<br>*Metallura tyrianthina*<br>*Phaethornis striigularis*<br>*Saucerottia castaneiventris*<br>*Schistes geoffroyi*<br>*Thalurania colombica*<br>*Thalurania furcata*<br>*Uranomitra franciae*<br>*Urosticte benjamini*	Lifestyle: Aerial (species spends much of the time in flight, and hunts or forages predominantly on the wing).<br>Habitat type: Dense Forest<br>Mass: 3-5.2 gr.<br>Beak length (Culmen): 34-104 mm<br>Beak width: 1.9-3.1 mm.<br>Wing lenght: 39.1-56.8 mm.<br>Secondary feather length: 13.1-21.3 mm
Group 2	*Aglaiocercus kingii*<br>*Colibri thalassinus*<br>*Polyerata amabilis*	Lifestyle: Aerial (species spends much of the time in flight, and hunts or forages predominantly on the wing).<br>Habitat type: Dense and semi-open forest<br>Mass: 4.3-5.9 gr.<br>Beak length (Culmen): 20.6-23.7 mm<br>Beak width: 2-2.5 mm.<br>Wing length: 52.3-62.6 mm.<br>Secondary feather length: 18.3-23.6 mm
Group 3	*Anthracothorax nigricollis*<br>*Chrysuronia goudoti*<br>*Saucerottia cyanifrons*<br>*Saucerottia saucerottei*	Lifestyle: Aerial (species spends much of the time in flight, and hunts or forages predominantly on the wing).<br>Habitat type: Semi-open habitats (shrubland, woodland)<br>Mass: 194-408 gr.<br>Beak length (Culmen): 26.1-36.3 mm<br>Beak width: 9.9-16.5 mm.<br>Wing length: 184.8-308.3 mm.<br>Secondary feather length: 99.5-192.1 mm
Group 4	*Amazilia versicolor*<br>*Discosura conversii*<br>*Klais guimeti*<br>*Ocreatus underwoodii*	Lifestyle: Aerial (species spends much of the time in flight, and hunts or forages predominantly on the wing).<br>Habitat type: Dense and semi-open forest<br>Mass: 2.6-4.1 gr.

**Nectarivorous functional group**	**Species**	**Traits description**
	Ramphomicron microrhynchum	Beak length (Culmen): 14.4-19.1 mm<br>Beak width: 1.3-1.8 mm.<br>Wing length: 42.2-51 mm.<br>Secondary feather length: 13-19 mm
Group 5	*Chaetocercus mulsant*<br>*Philodice mitchellii*	Lifestyle: Aerial (species spends much of the time in flight, and hunts or forages predominantly on the wing).<br>Habitat type: Dense and semi-open forest<br>Mass: 3.1-3.9 gr.<br>Beak length (Culmen): 21-17.4 mm<br>Beak width: 1.4-1.5 mm.<br>Wing length: 35.7-39.2 mm.<br>Secondary feather length: 12.3-13.5 mm
Group 6	*Campylopterus falcatus*<br>*Chalybura urochrysia*<br>*Florisuga mellivora mellivora*<br>*Glaucis hirsutus*<br>*Heliodoxa jacula*<br>*Heliodoxa rubinoides*<br>*Lafresnaya lafresnayi*<br>*Threnetes ruckeri*	Lifestyle: Aerial (species spends much of the time in flight, and hunts or forages predominantly on the wing).<br>Habitat type: Dense forest<br>Mass: 5.2-8.1 gr.<br>Beak length (Culmen): 23-33.4 mm<br>Beak width: 2.3-3.2 mm.<br>Wing length: 56.6-69.3 mm.<br>Secondary feather length: 21-25.6 mm
Group 7	*Chlorestes julie*	Lifestyle: Aerial (species spends much of the time in flight, and hunts or forages predominantly on the wing).<br>Habitat type: Dense forest<br>Mass: 3.3 gr.<br>Beak length (Culmen): 16 mm<br>Beak width: 1.2 mm.<br>Wing length: 43.1 mm.<br>Secondary feather length: 30 mm
Group 8	*Chlorostilbon gibsoni*<br>*Chlorostilbon poortmani*	Lifestyle: Aerial (species spends much of the time in flight, and hunts or forages predominantly on the wing).<br>Habitat type: Semi-open forest and woodland<br>Mass: 2.6-3.3 gr.<br>Beak length (Culmen): 16.9-19.2 mm<br>Beak width: 1.5-1.9 mm.<br>Wing length: 41.2-49.4 mm.<br>Secondary feather length: 13.7-15.5 mm
Group 9	*Coereba flaveola*	Lifestyle: Insessorial (species spends much of the time perching above the ground, in branches of trees, or on other raised substrates).<br>Habitat type: Shrubland<br>Mass: 10 gr.<br>Beak length (Culmen): 13.2 mm<br>Beak width: 3.6 mm.<br>Wing length: 54.6 mm.<br>Secondary feather length: 45.9 mm
Group 10	*Colibri coruscans*<br>*Phaeochroa cuvierii*<br>*Polytmus guainumbi*	Lifestyle: Aerial (species spends much of the time in flight, and hunts or forages predominantly on the wing).<br>Habitat type: Human-modified and grassland<br>Mass: 5.2-8.9 gr.<br>Beak length (Culmen): 24.2-29.1 mm<br>Beak width: 2.8-2.9 mm.<br>Wing length: 59.2-75.4 mm.<br>Secondary feather length: 21.6-28.5 mm

Nectarivorous functional group	Species	Traits description
Group 11	*Ensifera ensifera*	Lifestyle: Aerial (species spends much of the time in flight, and hunts or forages predominantly on the wing).<br>Habitat type: Forest<br>Mass: 11.2 gr.<br>Beak length (Culmen): 97.4 mm<br>Beak width: 2.5 mm.<br>Wing length: 76.3 mm.<br>Secondary feather length: 32.3 mm
Group 12	*Eutoxeres aquila*<br>*Heliodoxa imperatrix*	Lifestyle: Insessorial (species spends much of the time perching above the ground, in branches of trees, or on other raised substrates).<br>Habitat type: Forest<br>Mass: 8.8-10.6 gr.<br>Beak length (Culmen): 27.5-33.1 mm<br>Beak width: 3.1-3.6 mm.<br>Wing length: 70.5-73 mm.<br>Secondary feather length: 25.5-27.8 mm
Group 13	*Heliangelus exortis*	Lifestyle: Insessorial (species spends much of the time perching above the ground, in branches of trees, or on other raised substrates).<br>Habitat type: Forest<br>Mass: 4.6 gr.<br>Beak length (Culmen): 19.8 mm<br>Beak width: 1.9 mm.<br>Wing length: 60.8 mm.<br>Secondary feather length: 22.5 mm
Group 14	*Phaethornis guy*<br>*Phaethornis longirostris*<br>*Phaethornis superciliosus*<br>*Phaethornis syrmatophorus*	Lifestyle: Aerial (species spends much of the time in flight, and hunts or forages predominantly on the wing).<br>Habitat type: Dense and semi-open Forest<br>Mass: 5.8-6.3 gr.<br>Beak length (Culmen): 40.3-43.1 mm<br>Beak width: 2.7-3.3 mm.<br>Wing length: 58.4-61.2 mm.<br>Secondary feather length: 21-24.9 mm
Group 15	*Coeligena coeligena*<br>*Coeligena torquata*<br>*Phaethornis anthophilus*<br>*Phaethornis hispidus*<br>*Phaethornis yaruqui*	Lifestyle: Aerial (species spends much of the time in flight, and hunts or forages predominantly on the wing).<br>Habitat type: Dense Forest<br>Mass: 5-6.8 gr.<br>Beak length (Culmen): 34.4-45.6 mm<br>Beak width: 2.3-2.8 mm.<br>Wing length: 54.1-74.1 mm.<br>Secondary feather length: 21.7-29.2 mm

Functional safeguards for conservation: identifying climate change refugia for frugivorous and nectarivorous birds in a degraded area of Colombia

Fausto Sáenz-Jiménez, María Alejandra Parrado-Vargas, José F. González-Maya & Juan Emiro Carvajal-Cogollo

SUPPORTING INFORMATION
S4 TABLE

Supporting Information 4 (S2 Table). Shifts in the distribution area of all groups

Functional group	Scenarios																
	Current	CMCC-ESM2								MPI-ESM1-2-HR							
		SSP1 2.6 2021-2040	%	SSP1 2.6 2061-2080	%	SSP5 8.5 2021-2040	%	SSP5 8.5 2061-2080	%	SSP1 2.6 2021-2040	%	SSP1 2.6 2061-2080	%	SSP5 8.5 2021-2040	%	SSP5 8.5 2061-2080	%
Frugivores G1	123,989	115,957	-6	106,979	-14	111,364	-10	81,887	-34	115,995	-6	113,531	-8	115,074	-7	85,967	-31
Frugivores G1 (HFP)	81,382	75,499	-7	69,250	-15	72,114	-11	53,042	-35	75,671	-7	74,014	-9	75,074	-8	56,018	-31
Frugivores G2	213,183	219,503	3	223,301	5	222,077	4	235,390	10	221,299	4	225,669	6	222,909	5	233,147	9
Frugivores G2 (HFP)	118,885	123,574	4	127,297	7	126,163	6	138,945	17	125,827	6	129,976	9	127,184	7	138,257	16
Frugivores G3	141,674	137,954	-3	137,954	-3	129,696	-8	114,919	-19	135,385	-4	130,774	-8	133,744	-6	109,617	-23
Frugivores G3 (HFP)	90,620	89,428	-1	86,960	-4	84,429	-7	76,178	-16	87,928	-3	85,135	-6	87,216	-4	72,925	-20
Frugivores G4	208,711	220,049	5	221,080	6	201,744	-3	207,819	-0	213,256	2	213,188	2	217,743	4	207,228	-1
Frugivores G4 (HFP)	130,037	134,932	4	135,316	4	126,918	-2	129,340	-1	132,862	2	132,982	2	134,636	4	131,268	1
Frugivores G5	103,474	126,734	22	131,390	27	114,478	11	184,107	78	120,017	16	120,578	17	123,230	19	179,152	73
Frugivores G5 (HFP)	72,089	84,723	18	85,064	18	76,463	6	114,207	58	79,741	11	80,301	11	81,684	13	110,170	53
Frugivores G6	174,269	149,528	-14	125,145	-28	128,286	-26	92,569	-47	148,596	-15	142,065	-18	145,557	-16	88,790	-49
Frugivores G6 (HFP)	99,947	89,731	-10	78,763	-21	80,670	-19	56,196	-44	90,476	-9	88,425	-12	90,386	-10	59,750	-40
Frugivores G7	211,064	226,642	7	228,091	8	218,022	3	218,610	4	223,192	6	222,015	5	225,499	7	214,387	2
Frugivores G7 (HFP)	126,552	133,317	5	134,976	7	128,922	2	130,970	3	131,793	4	131,438	4	132,756	5	130,970	3
Frugivores G8	67,014	59,068	-12	55,102	-18	57,032	-15	46,807	-30	58,870	-12	53,357	-20	57,733	-14	32,486	-52
Frugivores G8 (HFP)	63,094	55,035	-13	50,950	-19	53,325	-15	42,915	-32	54,892	-13	49,603	-21	53,763	-15	29,296	-54
Frugivores G9	88,794	73,270	-17	65,814	-26	59,591	-33	47,512	-46	65,951	-26	52,294	-41	60,255	-32	33,520	-62
Frugivores G9 (HFP)	85,916	69,974	-19	61,691	-28	57,260	-33	43,525	-49	62,971	-27	49,796	-42	57,247	-33	30,207	-65
Frugivores G10	191,581	164,687	-14	143,576	-25	149,360	-22	102,984	-46	160,909	-16	154,983	-19	158,239	-17	103,983	-46
Frugivores G10 (HFP)	123,469	110,086	-11	97,395	-21	101,162	-18	67,503	-45	107,958	-13	104,641	-15	106,591	-14	70,369	-43
Frugivores G11	102,609	89,520	-13	70,624	-31	93,311	-9	46,339	-55	95,329	-7	108,925	6	98,191	-4	86,261	-16
Frugivores G11 (HFP)	69,992	62,270	-11	51,810	-26	66,239	-5	32,152	-54	67,928	-3	77,726	11	69,987	-0	61,837	-12
Frugivores G12	118,494	116,714	-2	107,450	-9	98,432	-17	74,737	-37	110,926	-6	103,585	-13	110,112	-7	102,844	-13
Frugivores G12 (HFP)	116,478	114,736	-1	105,480	-9	96,792	-17	73,182	-37	109,099	-6	101,897	-13	108,320	-7	101,336	-13
Nectarivores G1	162,338	151,824	-6	137,204	-15	137,273	-15	94,541	-42	149,284	-8	145,568	-10	147,703	-9	105,971	-35
Nectarivores G1 (HFP)	112,184	105,692	-6	95,366	-15	94,899	-15	62,719	-44	104,011	-7	101,571	-9	102,924	-8	71,964	-36
Nectarivores G2	90,346	86,230	-5	79,426	-12	80,274	-11	55,816	-38	85,555	-5	82,714	-8	84,578	-6	58,295	-35
Nectarivores G2 (HFP)	58,332	56,076	-4	52,073	-11	51,820	-11	37,027	-37	55,686	-5	53,823	-8	55,116	-6	38,413	-34

Functional group	Scenarios																
	Current	CMCC-ESM2								MPI-ESM1-2-HR							
		SSP1 2.6 2021-2040	%	SSP1 2.6 2061-2080	%	SSP5 8.5 2021-2040	%	SSP5 8.5 2061-2080	%	SSP1 2.6 2021-2040	%	SSP1 2.6 2061-2080	%	SSP5 8.5 2021-2040	%	SSP5 8.5 2061-2080	%
Nectarivores G3	205,330	181,599	-12	151,529	-26	161,301	-21	100,629	-51	182,243	-11	170,870	-17	177,094	-14	101,767	-50
Nectarivores G3 (HFP)	115,677	101,832	-12	87,693	-24	91,370	-21	63,338	-45	101,175	-13	94,580	-18	98,225	-15	62,774	-46
Nectarivores G4	128,452	135,376	5	134,988	5	105,249	-18	91,121	-29	128,753	0	121,254	-6	131,789	3	104,687	-19
Nectarivores G4 (HFP)	90,949	97,435	7	95,159	5	75,355	-17	68,016	-25	93,413	3	88,761	-2	95,908	5	82,089	-10
Nectarivores G5	96,668	90,305	-7	83,905	-13	86,201	-11	63,173	-35	90,589	-6	87,582	-9	89,539	-7	63,496	-34
Nectarivores G5 (HFP)	59,947	56,233	-6	52,438	-13	53,489	-11	40,116	-33	56,324	-6	54,384	-9	55,672	-7	39,827	-34
Nectarivores G6	202,154	199,250	-1	188,404	-7	179,496	-11	151,637	-25	195,454	-3	191,205	-5	195,675	-3	160,008	-21
Nectarivores G6 (HFP)	129,658	128,585	-1	122,889	-5	118,864	-8	100,312	-23	127,510	-2	125,360	-3	127,665	-2	110,888	-14
Nectarivores G7	36,866	34,313	-7	40,457	10	53,970	46	78,504	113	43,808	19	49,082	33	44,580	21	105,983	187
Nectarivores G7 (HFP)	21,563	19,479	-10	22,797	6	33,198	54	45,352	110	25,969	20	29,333	36	26,047	21	63,858	196
Nectarivores G8	146,984	165,368	13	161,968	10	153,850	5	186,856	27	151,946	3	139,170	-5	152,917	4	146,530	-0
Nectarivores G8 (HFP)	73,672	85,398	16	82,137	11	79,604	8	99,363	35	76,619	4	69,764	-5	77,058	5	74,871	2
Nectarivores G9	188,717	202,818	7	194,638	3	187,818	-0	115,305	-39	199,041	5	199,695	6	202,182	7	127,368	-33
Nectarivores G9 (HFP)	182,941	195,315	7	187,180	2	181,080	-1	110,529	-40	192,386	5	193,023	6	195,284	7	122,945	-33
Nectarivores G10	95,467	87,875	-8	79,709	-17	83,976	-12	59,091	-38	88,503	-7	85,855	-10	87,461	-8	62,484	-35
Nectarivores G10 (HFP)	91,030	83,707	-8	75,713	-17	79,905	-12	55,460	-39	84,317	-7	81,729	-10	83,300	-8	58,787	-35
Nectarivores G11	44,833	36,764	-18	34,072	-24	28,827	-36	15,707	-65	36,123	-19	29,009	-35	34,790	-22	10,630	-76
Nectarivores G11 (HFP)	29,787	25,616	-14	23,230	-22	22,293	-25	13,435	-55	25,538	-14	21,775	-27	24,714	-17	9,664	-68
Nectarivores G12	67,320	60,106	-11	53,432	-21	48,132	-29	33,157	-51	55,811	-17	49,824	-26	56,527	-16	35,491	-47
Nectarivores G12 (HFP)	50,429	44,456	-12	37,480	-26	36,172	-28	23,666	-53	42,185	-16	38,152	-24	43,027	-15	25,421	-50
Nectarivores G13	36,587	28,659	-22	25,743	-30	20,416	-44	12,252	-67	27,320	-25	20,305	-45	25,853	-29	10,459	-71
Nectarivores G13 (HFP)	27,283	22,373	-18	19,760	-28	18,053	-34	11,021	-60	21,711	-20	17,400	-36	20,929	-23	9,406	-66
Nectarivores G14	127,291	123,488	-3	112,193	-12	108,306	-15	82,125	-35	118,576	-7	114,366	-10	115,512	-9	83,088	-35
Nectarivores G14 (HFP)	91,603	87,582	-4	78,613	-14	76,975	-16	57,242	-38	85,772	-6	82,973	-9	84,375	-8	59,401	-35
Nectarivores G15	93,723	86,901	-7	79,560	-15	82,697	-12	58,880	-37	87,198	-7	84,099	-10	86,191	-8	59,340	-37
Nectarivores G15 (HFP)	58,643	54,705	-7	50,357	-14	52,206	-11	37,563	-36	54,846	-6	53,110	-9	54,276	-7	38,179	-35

Functional safeguards for conservation: identifying climate change refugia for frugivorous and nectarivorous birds in a degraded area of Colombia

Fausto Sáenz-Jiménez, María Alejandra Parrado-Vargas, José F. González-Maya & Juan Emiro Carvajal-Cogollo

SUPPORTING INFORMATION
S3 TABLE

Supporting Information 5 (S3 Table). Shifts in average elevation. Values of the functional groups with the greatest variation are underlined.

Scenario	Elevation	Frugivores											
		**1**	**2**	**3**	**4**	**5**	**6**	**7**	**8**	**9**	**10**	**11**	**12**
**Current**	**Average**	1845	524	1358	594	391	503	659	1541	1015	959	847	899
	**Min**	613	1	26	4	2	1	7	767	41	6	18	23
	**Max**	3630	2572	3414	2582	1162	1742	2742	2629	2552	3039	1922	2143
**SSP5 8.5 2080**	**Average**	2443	532	1940	748	383	1725	1344	2386	2262	2059	1590	1877
	**Min**	1405	1	529	9	1	38	2	1620	1171	919	664	854
	**Max**	3770	3014	3670	2742	1253	3039	2686	3117	3088	3656	3591	3067
**Average difference**		**598**	**8**	**582**	**154**	**-8**	**1222**	**685**	**845**	**1247**	**1100**	**743**	**978**

Scenario	Elevation	Nectarivores														
		**1**	**2**	**3**	**4**	**5**	**6**	**7**	**8**	**9**	**10**	**11**	**12**	**13**	**14**	**15**
**Current**	**Average**	1239	2100	645	1772	2017	988	252	390	483	2231	2880	1471	2621	1401	2187
	**Min**	20	1035	1	16	996	6	3	1	1	1142	2112	553	1758	126	1090
	**Max**	3414	3486	2645	3656	3574	3414	653	2126	2561	3714	3735	2626	3690	3040	3656
**SSP5 8.5 2080**	**Average**	2370	2863	2069	2143	2690	1493	482	553	1670	2832	3338	2304	3328	2188	2822
	**Min**	996	2001	56	38	1806	25	3	1	3	2003	2721	1626	2652	1062	1964
	**Max**	3884	3884	3630	3884	3770	3630	1068	2686	3574	3884	3972	3162	4003	3630	3854
**Average difference**		**1131**	**763**	**1424**	**371**	**673**	**505**	**230**	**163**	**1187**	**601**	**458**	**833**	**707**	**787**	**635**
**TOTAL AVERAGE**	**690**															
